# O-GlcNAc modification of MYPT1 modulates lysophosphatidic acid–induced cell contraction in fibroblasts

**DOI:** 10.1016/j.jbc.2021.100800

**Published:** 2021-05-19

**Authors:** Murielle M. Morales, Nichole J. Pedowitz, Matthew R. Pratt

**Affiliations:** 1 Department of Biological Sciences, University of Southern California, Los Angeles, California, USA; 2 Department of Chemistry, University of Southern California, Los Angeles, California, USA

**Keywords:** O-GlcNAc, lysophosphatidic acid, actomyosin, fibroblast, cell contraction, ATCC, American Type Culture Collection, DMEM, Dulbecco's modified Eagle's medium, DMSO, dimethyl sulfoxide, FCS, fetal calf serum, LPA, lysophosphatidic acid, MLC, myosin light chain, MYPT1, myosin-phosphatase target subunit 1, OGA, *O*-GlcNAcase, OGT, *O*-GlcNAc transferase, S1P, sphingosine-1-phosphate

## Abstract

Thousands of proteins have been found to be modified by *O*-GlcNAc, a common glycosylation modification of serine and threonine residues throughout the cytosol and nucleus. *O*-GlcNAc is enzymatically added and removed from proteins, making it a potential dynamic regulator of cell signaling. However, compared with other posttranslational modifications like phosphorylation, relatively few *O*-GlcNAc-regulated pathways have been discovered and biochemically characterized. We previously discovered one such pathway, where *O*-GlcNAc controls the contraction of fibroblasts initiated by the signaling lipid sphingosine-1-phosphate. Specifically, we found that *O*-GlcNAc modification of the phosphatase MYPT1 maintains its activity, resulting in dephosphorylation and deactivation of the myosin light chain of the actinomyosin complex. Another signaling lipid that leads to contraction of fibroblasts is lysophosphatidic acid, and this signaling pathway also converges on MYPT1 and actinomyosin. We therefore rationalized that *O*-GlcNAc would also control this pathway. Here, we used a combination of small molecule inhibitors, 2D and 3D cell cultures, and biochemistry to confirm our hypothesis. Specifically, we found that *O*-GlcNAc levels control the sensitivity of mouse and primary human dermal fibroblasts to lysophosphatidic acid–induced contraction in culture and the phosphorylation of MLC and that MYPT1 *O*-GlcNAc modification is responsible. These findings further solidify the importance of *O*-GlcNAc in regulating the biology of fibroblasts in response to procontractile stimuli.

Cells and tissues convert a small percentage of circulating glucose to uridine diphosphate *N*-acetylglucosamine (UDP-GlcNAc) through the hexosamine biosynthetic pathway (1, 2). UDP-GlcNAc then serves as both a metabolic intermediate for the biosynthesis of other sugar donors and the substrate for a variety of glycosyltransferases. One such enzyme, *O*-GlcNAc transferase (OGT) (3, 4, 5), has an interesting property where its activity and selection of protein substrates are dependent on UDP-GlcNAc levels (6, 7). This sets up the resulting posttranslational modification *O*-GlcNAc (Fig. 1*A*) as a sensor of glucose abundance and metabolism (8, 9, 10). *O*-GlcNAc modifications are also dynamic, as they can be removed by an opposing enzyme termed *O*-GlcNAcase (OGA) (4, 5). These properties, combined with the fact that *O*-GlcNAc occurs on serine and threonine residues that can also be occupied by phosphorylation (11), make *O*-GlcNAc a potentially critical signaling regulator. Of note, overall *O*-GlcNAc levels are altered in several human diseases. For example, *O*-GlcNAc levels are lower in patients with Alzheimer’s disease compared with healthy age-matched individuals (12, 13, 14, 15). In contrast, the modification levels are elevated in every type of cancer examined compared with healthy tissue (16, 17, 18). In diabetes, excessive concentrations of circulating glucose during hyperglycemia drive increased UDP-GlcNAc concentrations giving higher *O*-GlcNAc levels (19, 20, 21, 22). Of importance, alterations in *O*-GlcNAc levels have been found to alter signaling pathways that contribute to disease phenotypes, often through the interplay with phosphorylation. For example, increased *O*-GlcNAc modification of insulin receptor substrate 1 (IRS-1) and the kinase ATK results in less phosphorylation of these proteins and insulin resistance (23). Evidence from human genetics also supports an important role for increased *O*-GlcNAc in the development of diabetes. Specifically, polymorphisms in the gene encoding OGA result in an increased risk for type II diabetes and a lower age of onset (24). Despite these and a handful of other findings, we believe that multiple additional signaling pathways are likely altered by *O*-GlcNAc given its widespread modification of over 1000 proteins (25).

**Figure 1:**
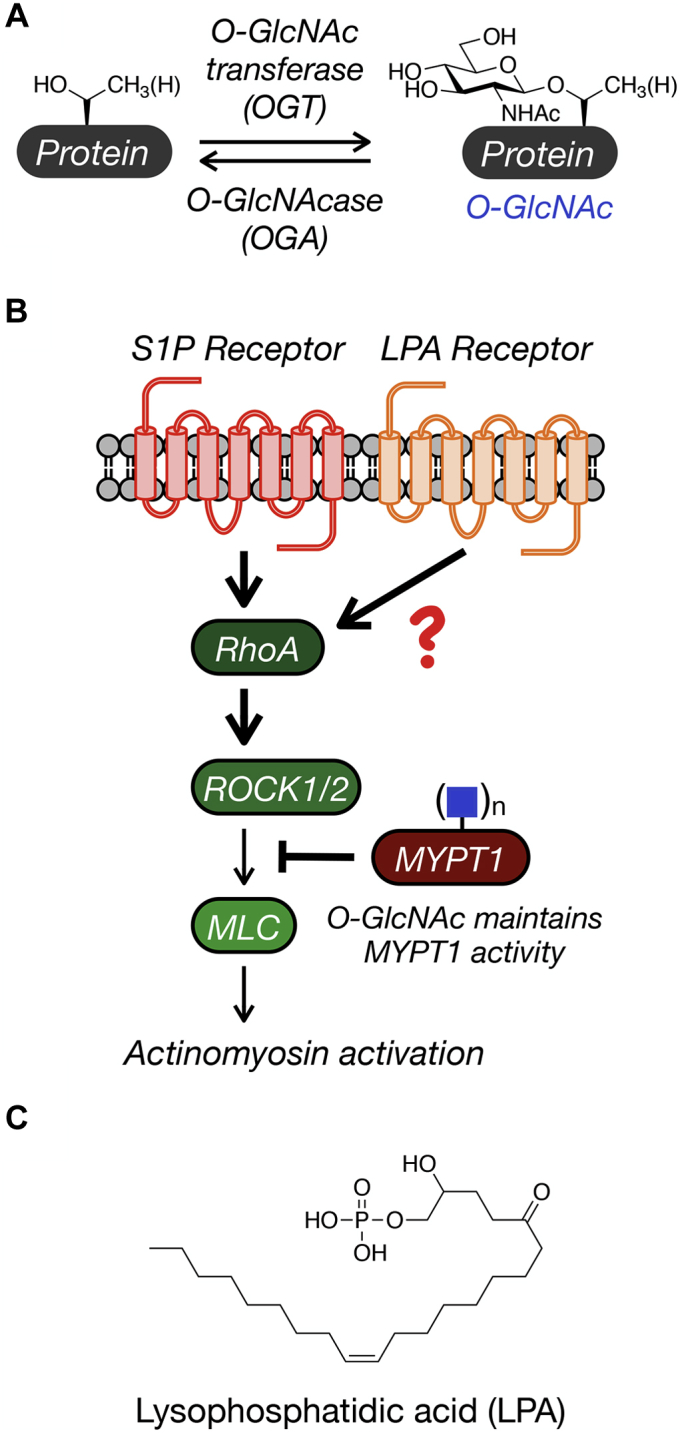
***O*-GlcNAc and contractile signaling.***A*, *O*-GlcNAc is the dynamic addition of *N*-acetylglucosamine to serine and threonine side chains of intracellular proteins. *B*, *O*-GlcNAc modification of the phosphatase MYPT1 opposes procontractile signaling. We previously discovered that *O*-GlcNAc on MYPT1 maintains its activity to block sphingosine-1-phosphate (S1P) signaling to limit actinomyosin activation and cell contraction. Here, we test if the same is true of lysophosphatidic acid (LPA). *C*, structure of LPA.

Recently, we discovered one such signaling pathway initiated by the lipid sphingosine-1-phosphate (S1P) (26). S1P is an agonist of a family of G-protein-coupled receptors, the S1P receptors (27, 28, 29, 30, 31, 32), resulting in the potential activation of a variety of downstream signaling pathways that regulate inflammation, cell motility, differentiation, etc. (33, 34). One of these pathways depicted in Figure 1*B* involves the activation of the GTPase RhoA and its associated kinase ROCK. ROCK can then phosphorylate myosin light chain (MLC), turning on the myosin motor and remodeling the actin cytoskeleton through a superstructure known as the actinomyosin complex (35). This pathway is opposed by a phosphatase holoenzyme consisting of the catalytic domain PP1C and the myosin-phosphatase target subunit 1 (MYPT1), which is responsible for dephosphorylating MLC, effectively turning off the myosin motor (36, 37). To ensure proper activation of the pathway, ROCK can also phosphorylate and deactivate MYPT1, thereby pushing on the gas and cutting the brakes simultaneously (38, 39). We found that *O*-GlcNAc modification of MYPT1 resists ROCK-mediated phosphorylation and that changing the *O*-GlcNAc levels on MYPT1 altered the sensitivity of fibroblasts to S1P-mediated actin contraction.

Here, we examine whether MYPT1 *O*-GlcNAc modification might also play a role in lysophosphatidic acid (LPA, Fig. 1*C*) signaling. LPA is biosynthesized from more common lipids like phosphatidylcholine, phosphatidylserine, and phosphatidylethanolamine (40). As shown in Figure 1*B*, LPA activates its own set of G-protein-coupled receptors and can signal into the same RhoA/ROCK/MLC pathway (40, 41, 42, 43, 44). We therefore hypothesized that *O*-GlcNAc levels would play a similar role in controlling the sensitivity of fibroblasts to LPA-induced actin contraction. To test this hypothesis, we first used a combination of small molecule inhibitors and growth conditions in different glucose concentrations to show that overall *O*-GlcNAc levels do indeed control LPA-mediated contraction in 2D cultures of NIH3T3 fibroblasts. We then used a MYPT1 *O*-GlcNAc-deficient mutant, MYPT1Δ, to confirm that this phenotype is due to MYTP1 *O*-GlcNAc modification. Finally, we show that *O*-GlcNAc controls LPA-mediated contraction of primary human dermal fibroblasts in 3D collagen cultures. S1P and LPA are found at reasonable concentrations (micromolar) in wound fluid where they promote fibroblast hepatotaxis, differentiation, and contraction during the wound healing process. We believe that these results further support a potential role for *O*-GlcNAc, particularly in diabetic nonhealing where *O*-GlcNAc levels are chronically increased.

## Results

### Global *O*-GlcNAc modification levels regulate fibroblast contraction in response to LPA

**Figure 2:**
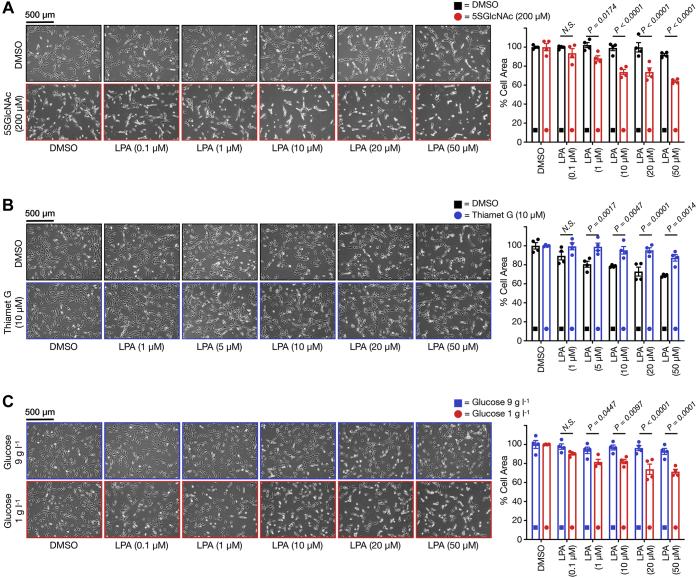
***O*-GlcNAc levels control the sensitivity of fibroblasts to lysophosphatidic acid (LPA)-mediated contraction in 2D culture.***A*, treatment with the OGT inhibitor 5SGlcNAc increases the sensitivity of NIH3T3 cells to LPA-induced contraction. Cells were treated with either DMSO or 5SGlcNAc (200 μM) before addition of the indicated concentrations of LPA. *B*, treatment with Thiamet G, an inhibitor that increases *O*-GlcNAc, renders NIH3T3 cells more resistant to LPA-induced contraction. Cells were treated with either DMSO or Thiamet G (10 μM) before addition of the indicated concentrations of LPA. *C*, glucose concentration in the medium controls the sensitivity of NIH3T3 cells to LPA-induced cell contraction. Cells were cultured in two concentrations of glucose before addition of the indicated concentrations of LPA. In all experiments, the contraction phenotype was then visualized using bright-field microscopy and quantitated. Results are the mean ± SEM of the relative culture plate area taken up by cells in four randomly selected frames (n = 4). Statistical significance was determined using a two-way ANOVA test followed by Sidak’s multiple comparisons test.

To determine whether overall *O*-GlcNAc levels alter the sensitivity of fibroblasts to LPA-induced contraction, we took advantage of two small molecule inhibitors: 5SGlcNAc (OGT inhibitor) (45) and Thiamet-G (OGA inhibitor) (46). Specifically, we first treated NIH3T3 mouse fibroblasts in 2D cell culture with 5SGlcNAc (200 μM) for 16 h, resulting in a loss of *O*-GlcNAc modification compared with the DMSO vehicle (Fig. S1*A*). After this length of time, we added a range of LPA concentrations (0–50 μM) and examined the extent of cell contraction after 30 min (Fig. 2*A*). As expected, we observed notable contraction on the culture plate for cells treated with higher concentrations of LPA regardless of their *O*-GlcNAc modification status. We can quantitate this amount of contraction by measuring how much space the cells take up on the culture plate before the addition of LPA to the space they occupy at the end of the assay. Using this method, we found that the cells pretreated with 5SGlcNAc showed significantly more contraction at lower LPA concentrations compared with those with normal *O*-GlcNAc levels. Next, we performed the opposite experiment by pretreatment of the NIH3T3 fibroblasts with Thiamet-G (10 μM) for 20 h, yielding increased *O*-GlcNAc levels (Fig. S1*A*), followed by LPA treatment. In this case, we observed less concentration from the cells treated with Thiamet-G (Fig. 2*B*). Of importance, these results were reproducible in a completely separate biological replicate (Fig. S2, *A* and *B*). Taken together these data confirm that *O*-GlcNAc levels do indeed alter the sensitivity of NIH3T3 fibroblasts to LPA in a similar fashion to S1P.

As noted above, *O*-GlcNAc levels are associated with the concentration of glucose in the environment. Therefore, it is possible that changes in glucose levels or uptake may have an impact on LPA-sensitivity through correspondingly altered *O*-GlcNAc modifications. To test this possibility, we cultured NIH3T3 cells in either 1 or 9 g l^−1^ glucose for 48 h, resulting in different *O*-GlcNAc levels as visualized by Western blotting (Fig. S1*B*). We then treated the cells with LPA and again measured cellular contraction. Consistent with our small molecule inhibitor experiments, the cells cultured in lower glucose concentrations were significantly more sensitive to LPA-mediated contraction compared with the fibroblasts grown in high glucose (Fig. 2*C*). Once again, we found the same results in a separate biological replicate (Fig. S2*C*). These results indicate that physiologically relevant changes in circulating glucose, for example, during hyperglycemia in diabetes, have the potential to dynamically regulate LPA signaling in fibroblasts.

### Reduced *O*-GlcNAc levels promote myosin light chain phosphorylation upon LPA stimulation

**Figure 3:**
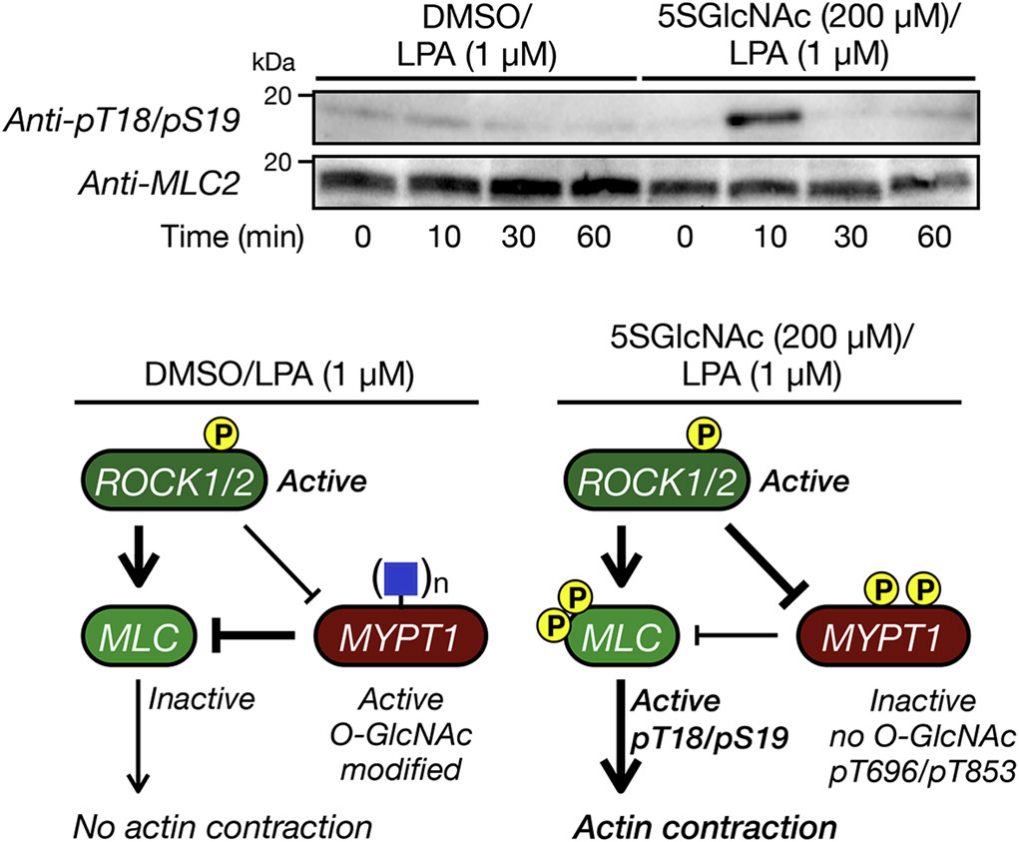
***O*-GlcNAc inhibits the activating phosphorylation of myosin light chain (MLC) upon lysophosphatidic acid (LPA) treatment.** NIH3T3 cells were treated with either dimethyl sulfoxide (DMSO) or 5SGlcNAc (200 μM) before addition of LPA (1 μM). Cells were lysed after the indicated lengths of time, and MLC phosphorylation was visualized by Western blotting.

We next examined whether reduction in *O*-GlcNAc levels results in a notable increase in MLC phosphorylation upon treatment with a relatively low concentration of LPA consistent with induction of the myosin motor and our observed contraction. Accordingly, we chose 1 μM LPA because it results in contraction under low *O*-GlcNAc conditions (5SGlcNAc treated) but very little under normal modification levels (Fig. 2*A*). We treated NIH3T3 fibroblasts with 5SGlcNAc (200 μM) or DMSO vehicle for 16 h, followed by LPA (1 μM). After different lengths of time, we collected the cells and examined the phosphorylation status of MLC by Western blotting (Fig. 3). In the case of the DMSO-treated cells we observed essentially no MLC phosphorylation at any of the timepoints, consistent with our model where MYPT1 *O*-GlcNAc modification maintains its activity and retains MLC in a dephosphorylated state. However, in the 5SGlcNAc-treated cells, we found a strong induction at MLC phosphorylation at 10 min that was subsequently lost after increased lengths of time. These results are highly consistent with the timing of cellular contraction and the fact that the NIH3T3 cells will eventually return to a relaxed state. Again, this supports the model built upon our published S1P data (26) where non-*O*-GlcNAc-modified MYPT1 can be phosphorylated and deactivated by ROCK (Fig. 3).

### MYPT1 *O*-GlcNAc modification controls LPA signaling to myosin

**Figure 4:**
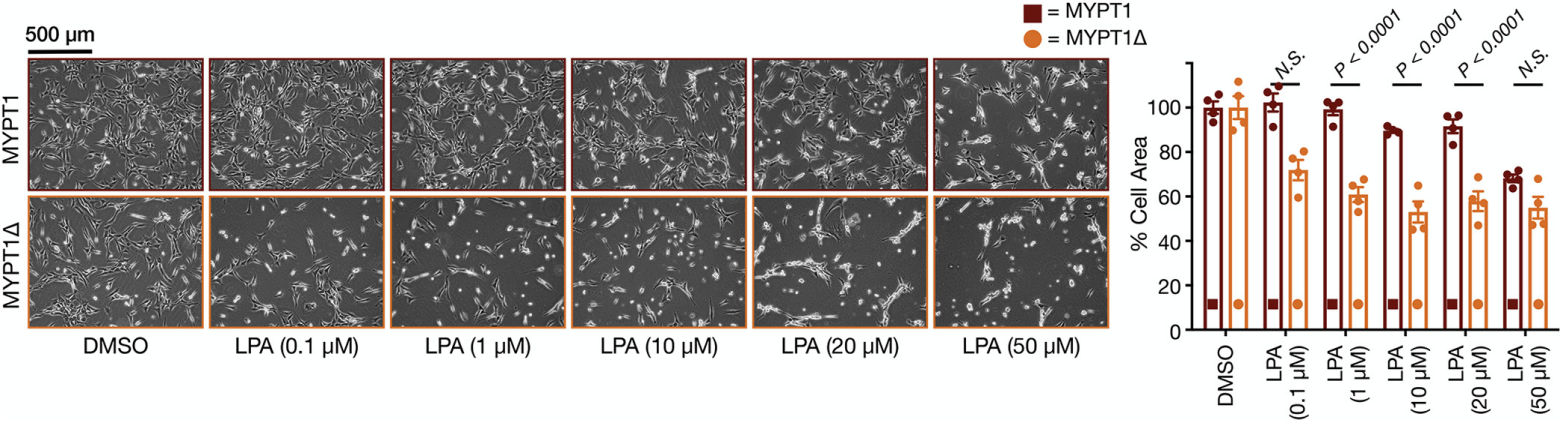
**Loss of MYPT1 *O*-GlcNAc modification sensitizes cells to lysophosphatidic acid (LPA)-mediated contraction in 2D culture.** NIH3T3 cells stably expressing either wildtype MYPT1 or the *O*-GlcNAc-deficient mutant MYPT1Δ were subjected to RNAi to knockdown the endogenous copy of MYPT1 before addition of the indicated concentrations of LPA. The contraction phenotype was then visualized using bright-field microscopy and quantitated. Results are the mean ± SEM of the relative culture plate area taken up by cells in four randomly selected frames (n = 4). Statistical significance was determined using a two-way ANOVA test followed by Sidak’s multiple comparisons test.

In our previous publication (26), we showed that MYPT1 is heavily *O*-GlcNAc modified at multiple sites in a serine/threonine-rich region located between residues 550 and 600. We also found that deletion of this entire region, yielding a protein we term MYPT1Δ, was required to notably reduce the overall *O*-GlcNAc levels but did not adversely affect MYPT1 phosphatase activity against MLC. However, MYPT1Δ was readily phosphorylated by ROCK upon S1P treatment resulting in sustained MLC phosphorylation and contraction. To determine if these same *O*-GlcNAc modifications on MYPT1 are responsible for controlling the sensitivity of cells to LPA-mediated contraction, we generated stable NIH3T3 cells expressing either FLAG-tagged MYPT1 or MYPT1Δ (Fig. S3*B*). Of note, these constructs utilized human MYPT1, allowing us to next use RNA interference (RNAi) to knockdown the endogenous copy of mouse MYPT1 (Fig. S3*B*). To confirm the efficiency of the RNAi, we used parent NIH3T3 cells because the stably expressed human MYPT1 or MYPT1Δ dominates the blot and prevents the knockdown of the endogenous MYPT1 from being detected. We then treated these cells with a range of LPA concentrations and once again measured the amount of cellular contraction in 2D (Fig. 4). Consistent with MYPT1 *O*-GlcNAc modification being directly important for inhibiting LPA signaling, we observed significantly more contraction in the MYPT1Δ cells compared with full-length MYPT1. Of importance, this result was repeated in two additional biological experiments (Fig. S4).

### *O*-GlcNAc levels control LPA-mediated contraction of primary cells in 3D culture

So far, we have confirmed that *O*-GlcNAc levels modulate the sensitivity of fibroblasts to LPA-mediated myosin activation and cell contraction in 2D culture and that MYPT1 *O*-GlcNAc modification is mostly likely responsible for this observation. However, NIH3T3 cells are immortalized mouse fibroblasts and the experimental conditions are on hard plastic. Therefore, our 2D contraction experiments are not completely physiologically relevant. To address this issue, we chose to next use 3D collagen matrices. Collagen matrices display mechanical and structural features that mimic connective tissue. Therefore, they are a physiologically relevant model system to investigate cell behavior. This is particularly true of fibroblast interactions that drive their motility and contraction (47, 48). In these experiments, acid-stabilized collagen is neutralized and mixed with fibroblasts before being allowed to solidify in a standard cell culture plate. The cells are then grown for 16 to 72 h. During this time, the cells and collagen fibers become aligned and actin stress fibers and focal adhesions form. The matrix is then released from the culture plate and procontractile factors, like LPA, can be added. The extent of 3D contraction can then be quantified by measuring the change in diameter of the collagen matrices.

**Figure 5:**
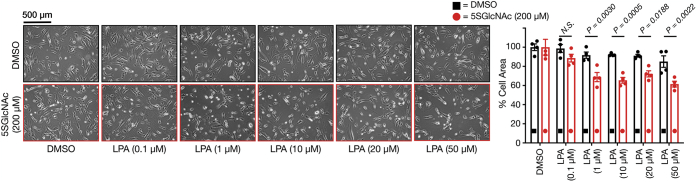
**Lowering *O*-GlcNAc levels increases the sensitivity of human dermal fibroblasts to S1P-induced cell contraction in 2D culture.** Cells were treated with dimethyl sulfoxide (DMSO) or 5SGlcNAc before the addition of the indicated concentrations of lysophosphatidic acid (LPA). The contraction phenotype was then visualized using bright-field microscopy and quantitated. Results are the mean ± SEM of the relative culture plate area taken up by cells in four randomly selected frames (n = 4). Statistical significance was determined using a two-way ANOVA test followed by Sidak’s multiple comparisons test.

**Figure 6:**
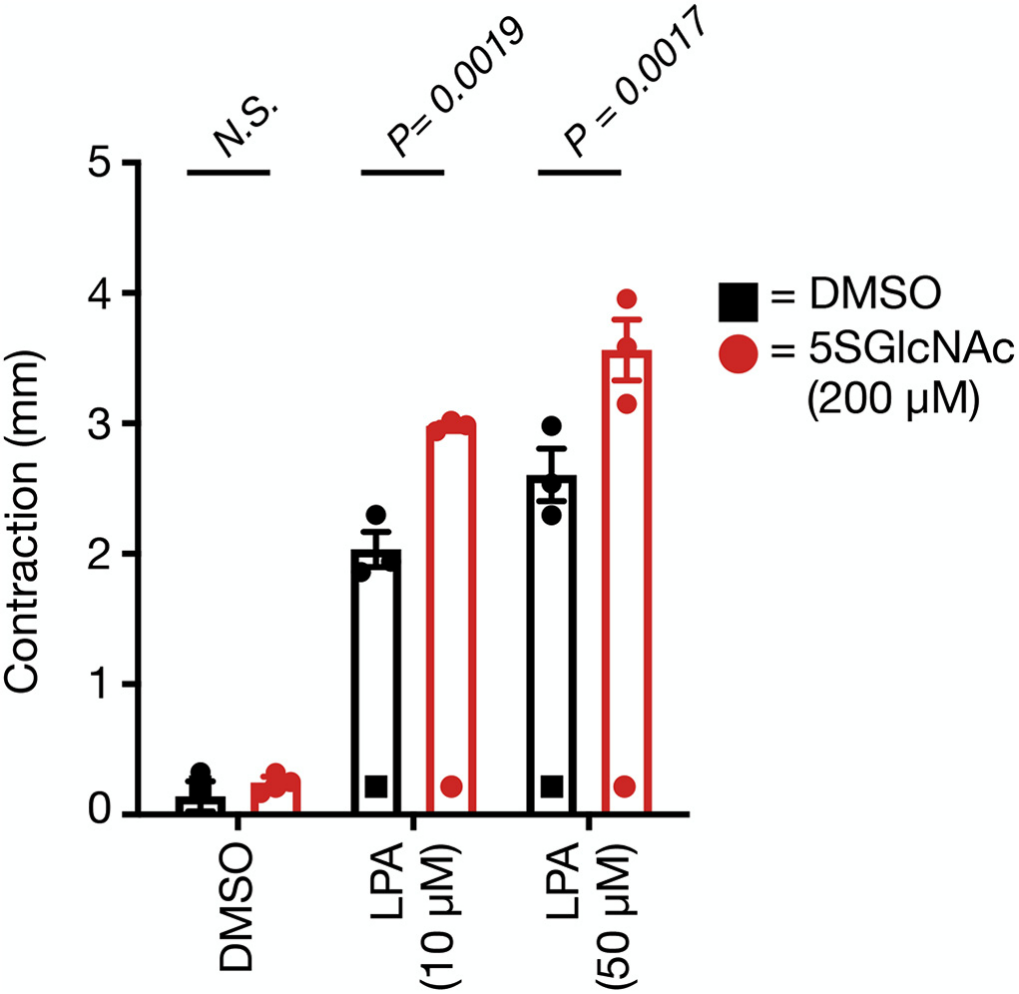
**Lowering *O*-GlcNAc increased the contraction of stressed collagen matrices by primary human fibroblasts in response to lysophosphatidic acid (LPA).** Contraction in millimeters was quantified as change in each matrix diameter from the initiation (t = 0 min) to termination (t = 30 min) of the assay. Results are the mean ± SEM of the millimeters contracted from three separate experiments. Statistical significance was determined using a two-way ANOVA test followed by Sidak’s multiple comparisons test.

To take advantage of this assay, we chose to use primary human dermal fibroblasts (ATCC BJ-5ta cells) in combination with 5SGlcNAc (200 μM), which decreased their overall *O*-GlcNAc levels as expected (Fig. S5*A*). We first performed a 2D contraction assay with these cells and found that they respond essentially the same as the NIH3T3 fibroblasts, where reduction in the *O*-GlcNAc levels sensitized them to LPA treatment (Fig. 5 and Fig. S5*B*). Finally, we generated 3D stressed collagen matrices with these dermal fibroblasts and allowed them to grow for 56 h before addition of 5SGlcNAc (200 μM) for another 16 h. We then released the matrices from the culture plate and added a range of LPA concentrations (0–50 μM) in triplicate. The matrices were then photographed (Fig. S6), and the diameter of the matrices was measured at t = 0 and 30 min using ImageJ. Analysis of the change in diameter from t = 0 to t = 30 min for each culture showed that LPA caused significantly more 3D contraction under low *O*-GlcNAc levels (Fig. 6). These results show that *O*-GlcNAc controls LPA-mediated fibroblast contraction in primary human cells in an established tissue culture model, strongly suggesting that our observations have physiological relevance.

## Discussion

Both S1P and LPA have been established as critical signaling molecules in fibroblast biology. For example, LPA induces fibroblast contraction in collagen matrices and induces fibroblast hepatotaxis in Boyden chambers (49, 50, 51, 52, 53). Our data demonstrate that *O*-GlcNAc may play an important role in controlling some of these signaling events through modulation of the Rho/ROCK/myosin system. Specifically, we found that *O*-GlcNAc levels control the sensitivity of fibroblasts to LPA in the medium, setting up this modification as a dimmer switch that regulates the concentration of LPA required to drive contraction. We also identified the critical *O*-GlcNAc modified protein to be the phosphatase MYPT1, where *O*-GlcNAc modification maintains its activity. Although not necessarily surprising, these results correlate well with our previous studies on S1P and indicate a general role for MYPT1 *O*-GlcNAc modification in the modulation of multiple signaling pathways that converge on MLC phosphorylation and activation. Taken together, we hypothesize that *O*-GlcNAc levels may contribute to deficits in wound healing, particularly in diabetes where the modification levels are increased. S1P and LPA are both found at reasonably high micromolar levels in wound fluid, and proper myosin activation is important for fibroblast migration into wounds, fibroblast differentiation, and wound remodeling through fibroblast contraction. Therefore, reduction of *O*-GlcNAc levels through small molecule inhibitors might be a strategy to rescue fibroblast biology in the treatment of chronically nonhealing wounds. Our results also suggest that *O*-GlcNAc could have a similar role in other cell types that use myosin activation and the actin cytoskeleton for their biology. For example, smooth muscle cells respond to a variety of peptide hormones (*e.g.*, endothenlin-1) (50, 54, 55) resulting in their contraction and the regulation of blood pressure. These pathways also converge at MLC and therefore are likely to be effected by MYPT1 *O*-GlcNAc modification.

In summary, we have further characterized our discovery of an important example of *O*-GlcNAc directly modulating a cell signaling pathway. Several outstanding questions remain regarding the biochemistry and exact mechanism by which *O*-GlcNAc affects MYPT1 activity. We previously demonstrated that *O*-GlcNAc prevents MYPT1’s phosphorylation and inactivation by ROCK and that this may occur by physically blocking the interaction between these two proteins (26). However, *O*-GlcNAc did not completely abolish this interaction, and we therefore cannot rule out more complicated mechanisms. For example, *O*-GlcNAc may also alter the baseline MYPT1 phosphatase activity or prevent phosphorylation by forcing an alternative protein confirmation. We are currently exploring these possibilities in a purified *in vitro* system. Despite these remaining questions, we believe that MYPT1 *O*-GlcNAc modification will play a significant role in multiple areas of biology, including other signaling pathways like cell division (56).

## Experimental procedures

### Cell culture

Mouse embryonic fibroblast cell line NIH3T3 (American Type Culture Collection [ATCC]) was propagated in Dulbecco's modified Eagle's medium (DMEM) (Genesee Scientific) supplemented with 10% fetal calf serum (FCS) (Atlanta Biologics). NIH3T3 cell lines stably expressing human FLAG-tagged MYPT1 or MYPT1Δ were grown in DMEM + 10% FCS supplemented with 250 ng ml^−1^ puromycin (1 mg ml^−1^ stock in H_2_O). Human dermal fibroblast cell line BJ-5ta (ATCC) was maintained in DMEM (Genesee Scientific) with 10% fetal bovine serum (Atlanta Biologics) and supplemented with 0.01 mg ml^−1^ hygromycin B (10 mg ml^−1^ in H_2_O). All cells were grown in a humidified incubator at 37 °C and 5% CO_2_ atmosphere.

### Synthesis of known small molecules

Known compounds Thiamet-G (46) and Ac45SGlcNAc (45) were synthesized according to literature procedures. Both were dissolved as 1000 X stocks in dimethyl sulfoxide (DMSO).

### Antibodies

Anti-MLC2 (3672S), anti-pT18/pS19 MLC (3674S), and anti-MYPT1 (2634S) were purchased from Cell Signaling Technology. Anti-RL2 (MA1-072) was purchased from ThermoFisher Scientific. Anti-β-actin (A5441) was purchased from MilliporeSigma. Horseradish peroxidase–conjugated secondary antibodies were purchased from Jackson ImmunoResearch. All antibodies were incubated in OneBlockTM Western-CL blocking buffer from Genesee Scientific (20-313).

### General Western blot procedure

Proteins were separated by SDS-PAGE before being transferred to a polyvinylidene difluoride membrane (Bio-Rad) using standard procedures. Western blots were blocked in OneBlockTM Western-CL blocking buffer (Genesee) for 1 h at r.t. before incubation with primary antibodies at 1:1000 dilution in fresh blocking buffer at 4 °C overnight. Blots were then washed in TBST (Cell Signaling, 3 × 10 min) and incubated with horseradish peroxidase–conjugated secondary antibody at 1:10,000 dilution in fresh blocking buffer for 1 h at r.t. Then blots were again washed in TBST (3 ×10 min). Blots were developed using ECL reagents (Bio-Rad) and the ChemiDoc XRS+ molecular imager (Bio-Rad). Uncropped blots can be found in Fig. S7.

### 2D contraction assay LPA concentration course

Mouse embryonic fibroblasts NIH3T3 or human dermal fibroblasts BJ-5ta cells were plated (1 × 105 cells) in six-well dishes and incubated for 8 h, followed by treatment with either DMSO vehicle, 5SGlcNAc (200 μM), or Thiamet-G (10 μM). After 16 or 20 h incubation with 5SGlcNAc or Thiamet-G, respectively, cells were treated with LPA (0.1–50 μM) for 30 min or 1 h. Each well was imaged through bright-field microscopy at 20× magnification using a Leica Microscope. Quantification of cell contraction was determined by taking the mean ± SEM of the culture plate area covered by cells in four randomly selected frames per well. Images were analyzed using Adobe Photoshop. Background pixels were selected using the Magic Wand tool and subtracted from total pixels. This value was then normalized using a control, untreated well allowing for quantification of the difference in cell area before and after contraction. Statistical significance was determined using a two-way ANOVA test followed by Sidak’s multiple comparisons test. Representative images for each treatment were selected from one of the four frames used for quantification.

### High/low glucose LPA concentration course

NIH3T3 cells were grown in media containing either high (9 g l^−1^) or low (1 g l^−1^) glucose concentrations for 24 h prior to being seeded as indicated above and incubated for an additional 24 h before treatment of cells with LPA (0.1–50 μM) for 30 min. Cell contraction was characterized as described.

### MYPT1 *versus* MYPT1Δ LPA concentration course

NIH3T3 cell lines expressing human FLAG-tagged MYPT1 or MYPT1Δ were transfected at 40% confluency with RNAi targeting mouse MYPT1 (transfection details described in a subsequent section). After 24 h, cells were plated (1 × 105 cells) in six-well dishes and incubated for 24 h. After 48 h total, cell lines were treated with LPA (0.1–50 μM) for 30 min. Contraction was characterized as stated above.

### Human dermal fibroblast concentration course

Primary human dermal fibroblasts, BJ-5ta, were plated (1 × 10^5^ cells) in six-well dishes for 8 h before treatment with either DMSO vehicle or 5SGlcNAc (200 μM) for 16 h. Cells were then treated with LPA (0.1–50 μM) for 1 h. Cell contraction was characterized as described.

### MLC activation Western blot time course

NIH3T3 cells were seeded 5 × 10^5^ cells in 10-cm dishes for 8 h before treatment with DMSO vehicle or 5SGlcNAc (200 μM). After 16 h incubation, low LPA (1 μM) was added to each plate per original treatment, and cells were incubated for 0, 10, 30, or 60 min. At each time point, the plate was placed on ice and the medium decanted, and the plate was rinsed with ice-cold PBS supplemented with PhoStop (5 mg ml^−1^) and harvested by scraping into precooled Falcon tubes. Cells were pelleted by centrifugation (5 min, 2000*g*, 4 °C) and resuspended in 4% SDS buffer (4% SDS, 150 mM NaCl, 50 mM TEA, pH 7.4) and lysed via tip sonication (3× 5 s on 5 s off). The lysate was centrifuged (10 min, 10,000*g*, rt), and supernatant was moved to a fresh tube. Protein concentration was determined by BCA Assay (Pierce, ThermoScientific), and gel samples were prepared at 2 mg ml^−1^ with appropriate volumes of 4% SDS buffer and 2× loading buffer (20% glycerol, 0.2% bromophenol blue, 1.4% β-mercaptoethanol, pH 6.8). A volume of 20 μl (40 μg) per sample was loaded per lane. Western blotting was performed as previously described in the general Western blot procedure above. Induction of MLC phosphorylation was visualized using anti-pT18/S19 MLC2. Expression of MLC2 was used as loading control.

### Generation of NIH3T3 cell lines stably expressing FLAG-tagged MYPT1 and MYPT1Δ mutants

FLAG-tagged MYPT1 and MYPT1Δ NIH3T3 cell lines were generated using the PiggyBac transposon vector system (System Biosciences). Briefly, NIH3T3 cells at 30% confluency in 10-cm dishes were cotransfected with 5 μg PiggyBac transposase promoter (plasmid ID: PB531A-1) and 10 μg of PiggyBac transposon plasmid pPB-CAG-IRES2-puro containing human MYPT1 or human MYPT1Δ550–600 (MYPT1Δ). One plate was transfected with 10 μg of pcDNA3 to serve as negative selection control. Twenty-four hours post transfection, plates were split 1:2 into normal growth medium (DMEM + 10% FCS) supplemented with 1 μg ml^−1^ puromycin (1 mg ml^−1^ stock in H_2_O). Cells were selected in puromycin for 3 days, at which point puromycin concentration was reduced to 250 ng ml^−1^. FLAG-tagged MYPT1 expression was confirmed by Western blotting against anti-FLAG and anti-MYPT1.

### Endogenous mouse MYPT1 knockdown time course with RNAi

NIH3T3 or FLAG-tagged MYPT1 stably expressed cell lines were seeded at 30% confluency in 10-cm dishes 24 h before transfection. RNAi (5 nmol) targeted against mouse MYPT1 or a scramble sequence was purchased from ThermoFisher Scientific (siRNA ID: s70342). A stock concentration of 10 μM was generated by diluting samples in 500 μl in nuclease-free H_2_O. Cells were transfected with 125 pmol (12.5 μl) of RNAi using Lipofectamine RNAiMAX transfection reagent (ThermoFisher Scientific) according to the manufacturer’s protocol. Plates were harvested at 0, 24, and 48 h. Knockdown efficiency was measured *via* Western blotting against MYPT1 according to the Western blot procedure described above.

### Endogenous mouse MYPT1 knockdown with RNAi

NIH3T3 cell lines expressing human MYPT1 or human MYPT1Δ were seeded at 30% confluency in 10-cm dishes 24 h before transfection. RNAi (5 nmol) targeting mouse MYPT1 was purchased from ThermoFisher Scientific (siRNA ID: s70342) and diluted in 500 μl of nuclease-free H_2_O to make a stock concentration of 10 μM. Cells were transfected with 125 pmol (12.5 μl) of RNAi using Lipofectamine RNAiMAX transfection reagent (ThermoFisher Scientific) according to the manufacturer’s protocol.

### Collagen gel contraction assay

The 3D contraction assay was performed using the two-step cell contraction assay kit purchased from Cell Biolabs (CBA-201) according to manufacturer’s protocol. BJ-5Ta cells were suspended in fresh medium at a confluency of 2 × 10^6^ cells per ml. Collagen stock solution was mixed with cell suspension at a 4:1 ratio, and 500 μl was plated per well in a 24-well dish. The cell–collagen mixture was then incubated at 37 °C for 1 h to allow collagen polymerization before adding 1 ml of medium to each well. After 56 h, wells were supplemented with either DMSO vehicle or 5SGlcNAc (200 μM) and incubated for an additional 16 h. The medium was then replaced with serum-free medium containing LPA (10, 50 μM), and contraction was initiated by gently releasing the matrices from the sides and bottom of the plate. Images were taken immediately after medium change at t = 0 and after 30 min using the ChemiDoc XRS+ molecular imager (Bio-Rad, Bio-Rad Image Lab 4.1). Contraction was measured using ImageJ (ImageJ 1.52q). Specifically, collagen matrix diameter was measured by taking the average of one horizontal (0°) and one vertical (90°) measurement of each matrix.

## Data availability

All data are contained in the article. Plasmid sequences are available from M. R. P. upon request at matthew.pratt@usc.edu.

## Supporting information

This article contains supporting information.

## Conflict of interest

The authors declare that they have no conflicts of interest with the contents of this article.
