# Supplementary material for: O-GlcNAc modification of MYPT1 modulates lysophosphatidic acid–induced cell contraction in fibroblasts
Source: J Biol Chem. 2021 May 19;296:100800. doi: 10.1016/j.jbc.2021.100800 (PMC8191289; doi:10.1016/j.jbc.2021.100800)
Supplement: Supplemental Figures S1–S7 [file mmc1.pdf]

O-GlcNAc modification of MYPT1 modulates lysophosphatidic acid (LPA)-induced cell contraction in fibroblasts.

Murielle M. Morales,[1] Nichole J. Pedowitz,[1] and Matthew R. Pratt[1,2,3]

[1]Departments of Chemistry and [2]Biological Sciences, University of Southern California, Los Angeles, CA 90089, United States

[3]Corresponding author: Matthew R. Pratt, matthew.pratt@usc.edu

Table of contents:

Figure S1. O-GlcNAc levels can be modulated by inhibitors and glucose concentration. **Page S2**
Figure S2. O-GlcNAc levels controls the sensitivity of fibroblasts to LPA-mediated contraction in 2D culture (biological replicates of data in Figure 2). **Page S3**
Figure S3. Characterization of MYPT1 and MYPT1Δ expressing cell lines. **Page S3**
Figure S4. Loss of MYPT1 O-GlcNAc modification sensitizes cells to LPA-mediated contraction in 2D culture (biological replicates of data in Figure 4). **Page S4**
Figure S5. O-GlcNAc controls LPA-mediated contraction of human dermal fibroblasts. **Page S5**
Figure S6. Raw images of the stressed collagen matrix assay. **Page S5**
Figure S7. Un-cropped blots. **Page S6**

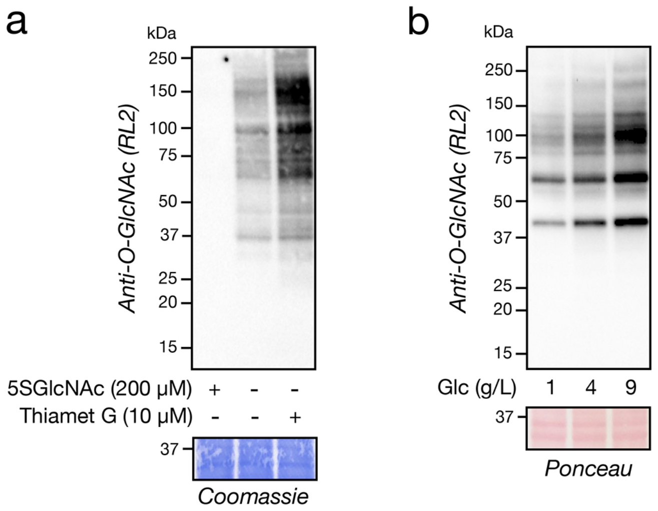

Figure S1. O-GlcNAc levels can be modulated by inhibitors and glucose concentration. a) NIH3T3 cells were treated with either 5SGlcNAc (200 µM, 16h), DMSO, or Thiamet G (10 µM, 20 h) before analysis of O-GlcNAc levels by western blotting. b) NIH3T3 cells were cultured in the indicated concentrations of glucose for 48 h before analysis of O-GlcNAc levels by western blotting.

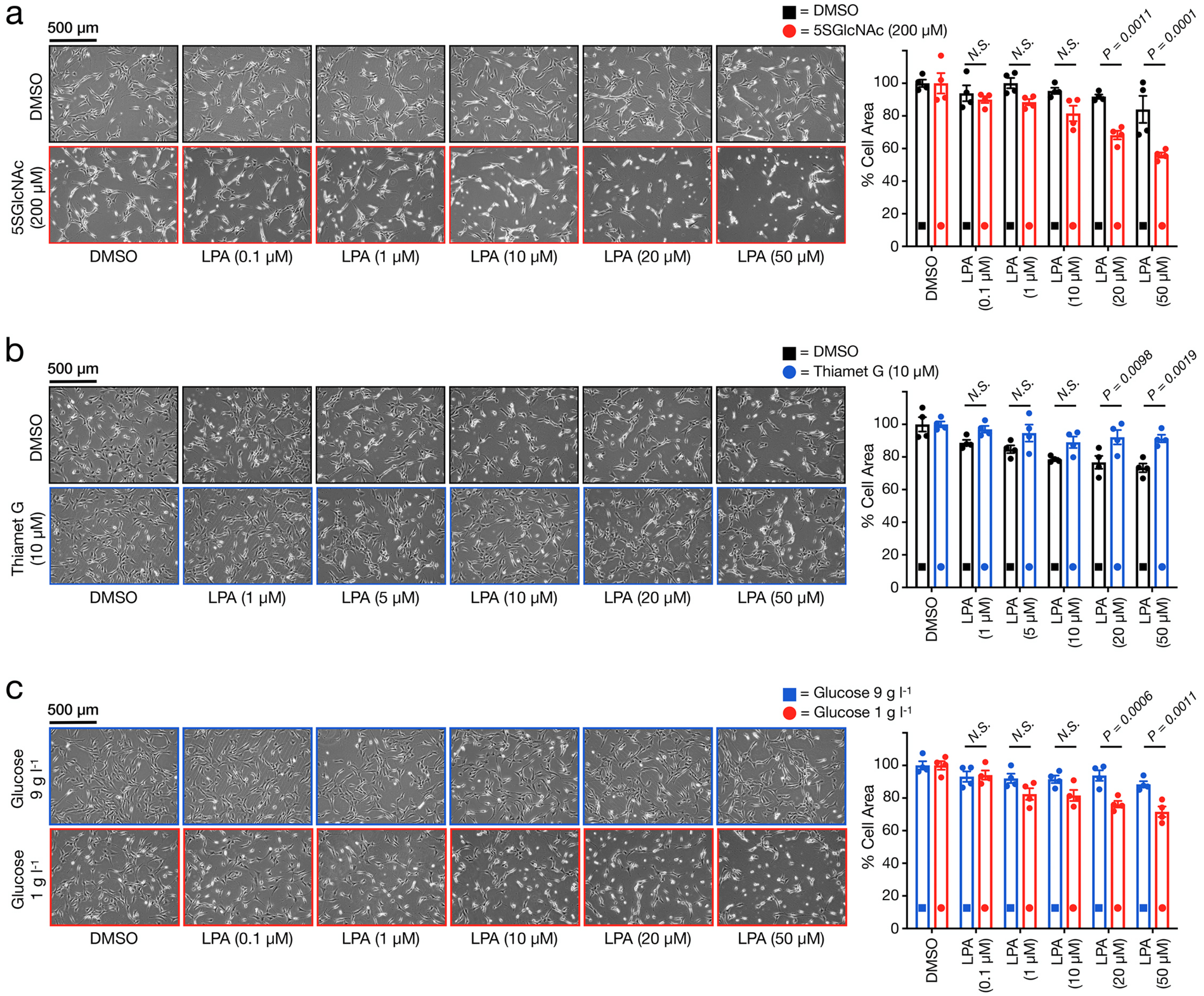

Figure S2. O-GlcNAc levels controls the sensitivity of fibroblasts to LPA-mediated contraction in 2D culture (biological replicates of data in Figure 2). a) Treatment with the OGT inhibitor 5SGlcNAc increases the sensitivity of NIH3T3 cells to LPA induced contraction. Cells were treated with either DMSO or 5SGlcNAc (200 µM) before addition of the indicated concentrations of LPA. b) Treatment with Thiamet G, an inhibitor that increases O-GlcNAc, renders NIH3T3 cells more resistant to LPA induced contraction. Cells were treated with either DMSO or Thiamet G (10 µM) before addition of the indicated concentrations of LPA. c) Glucose concentration in the media controls the sensitivity of NIH3T3 cells to LPA induced cell contraction. Cells were cultured in two concentrations of glucose before addition of the indicated concentrations of LPA. In all experiments, the contraction phenotype was then visualized using bright-field microscopy and quantitated. Results are the mean ± SEM of the relative culture plate area taken-up by cells in four randomly selected frames (n=4). Statistical significance was determined using a 2-way ANOVA test followed by Sidak's multiple comparisons test.

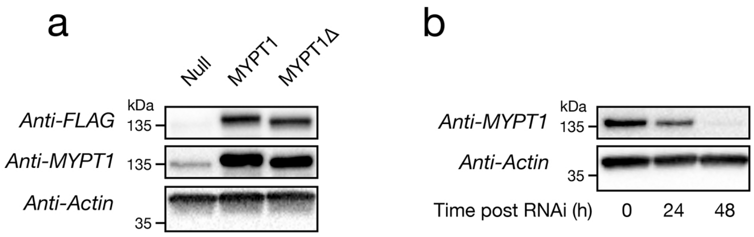

Figure S3. Characterization of MYPT1 and MYPT1Δ expressing cell lines. a) Generation of cells stably expressing MYPT1 or MYPT1Δ. NIH3T3 cells were stably transfected with either FLAG-tagged human MYTP1 constructs using the PiggyBac retrotransposon system before analysis by western blotting. b) RNAi efficiently knocks-down endogenous MYPT1. NIH3T3 cells were transfected with RNAi targeting mouse MYPT1 (Sigma) and protein levels were measured by western blotting after 24 and 48 h.

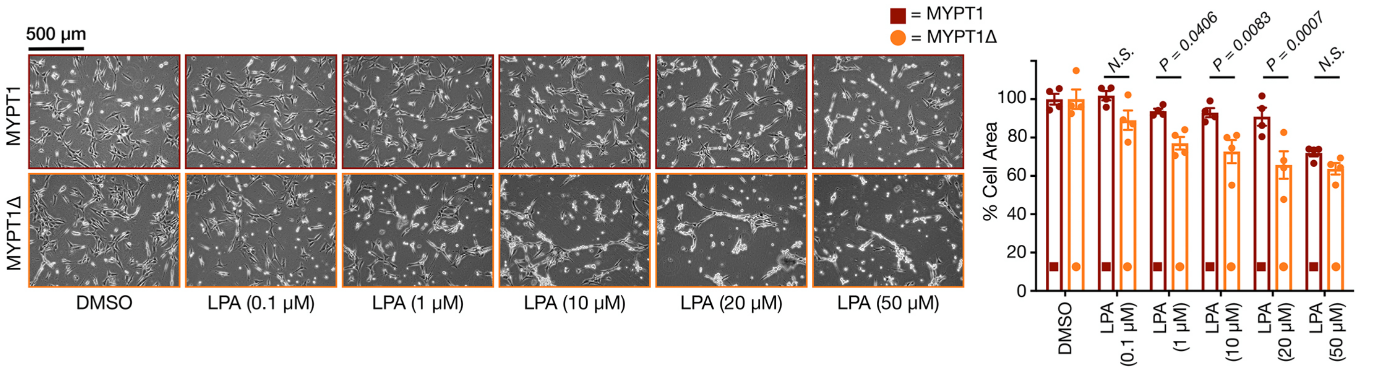

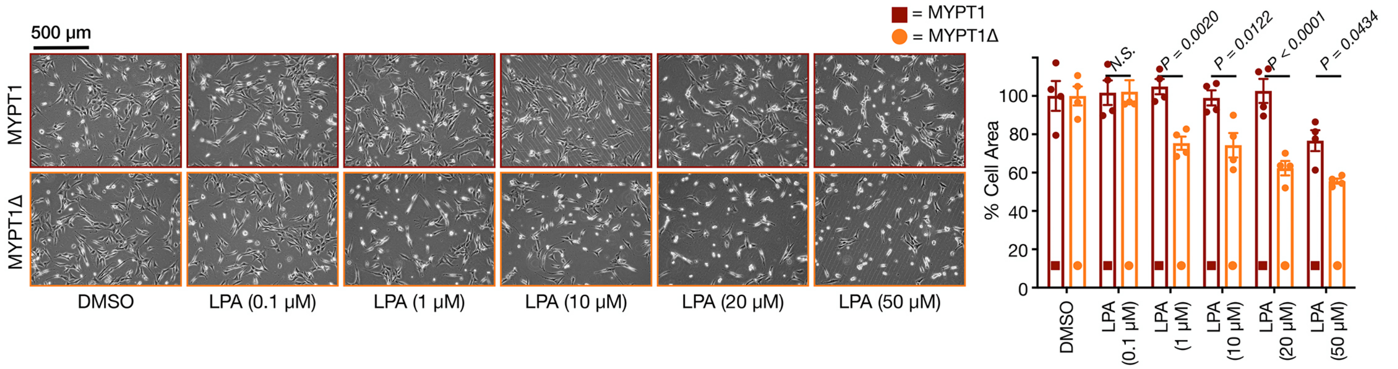

Figure S4. Loss of MYPT1 O-GlcNAc modification sensitizes cells to LPA-mediated contraction in 2D culture (biological replicates of data in Figure 4). NIH3T3 cells stably expressing either wild-type MYPT1 or the O-GlcNAc deficient mutant MYPT1Δ were subjected to RNAi to knockdown the endogenous copy of MYPT1 before addition of the indicated concentrations of LPA. The contraction phenotype was then visualized using bright-field microscopy and quantitated. Results are the mean ± SEM of the relative culture plate area taken-up by cells in four randomly selected frames (n=4). Statistical significance was determined using a 2-way ANOVA test followed by Sidak's multiple comparisons test.

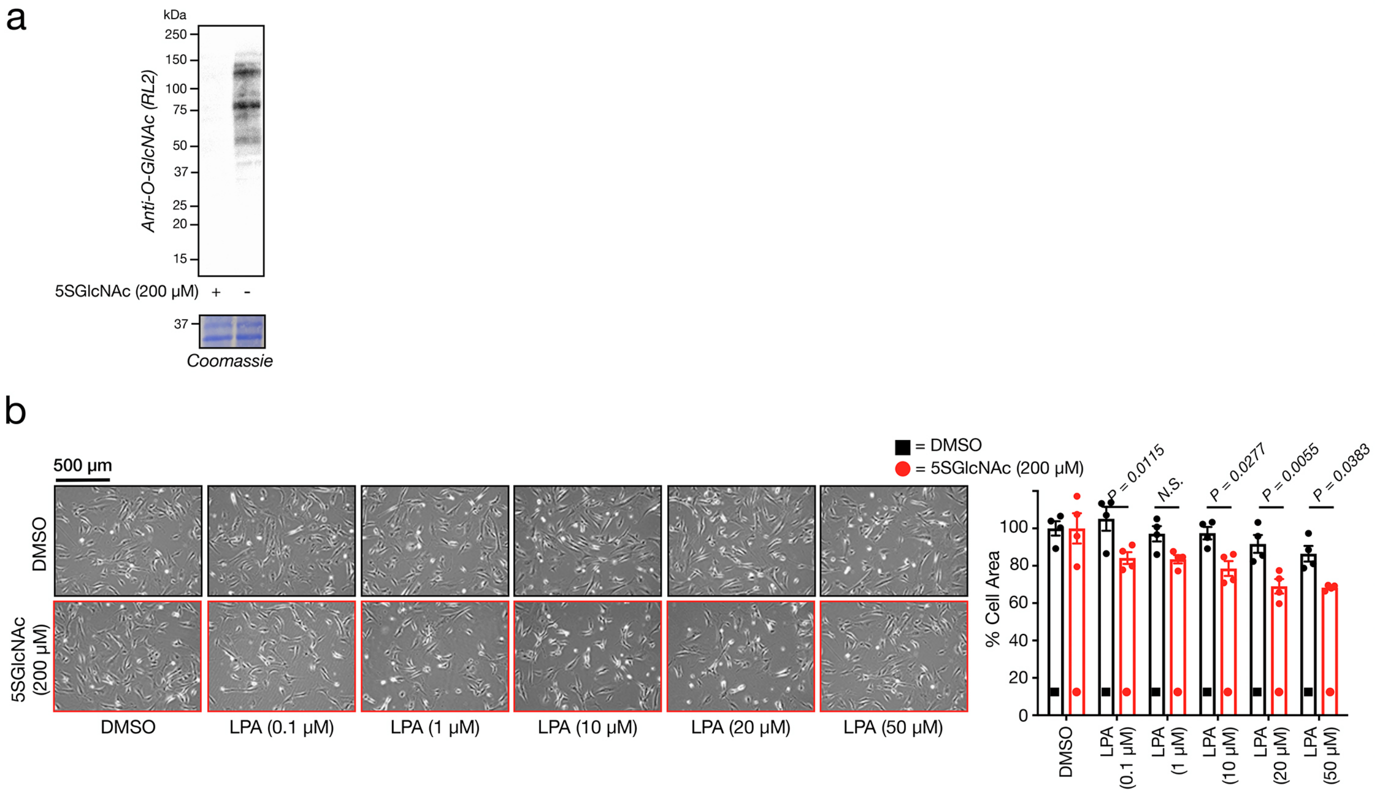

Figure S5. O-GlcNAc controls LPA-mediated contraction of human dermal fibroblasts. a) Human dermal fibroblasts were treated with 5SGlcNAc (200 μM) or DMSO before analysis of O-GlcNAc levels by western blotting. b) Lowering O-GlcNAc levels increases the sensitivity of human dermal fibroblasts to S1P induced cell contraction in 2D culture (biological replicate of data in Figure 5). Cells were treated with DMSO or 5SGlcNAc before the addition of the indicated concentrations of LPA. The contraction phenotype was then visualized using bright-field microscopy and quantitated. Results are the mean ± SEM of the relative culture plate area taken-up by cells in four randomly selected frames (n=4). Statistical significance was determined using a 2-way ANOVA test followed by Sidak's multiple comparisons test.

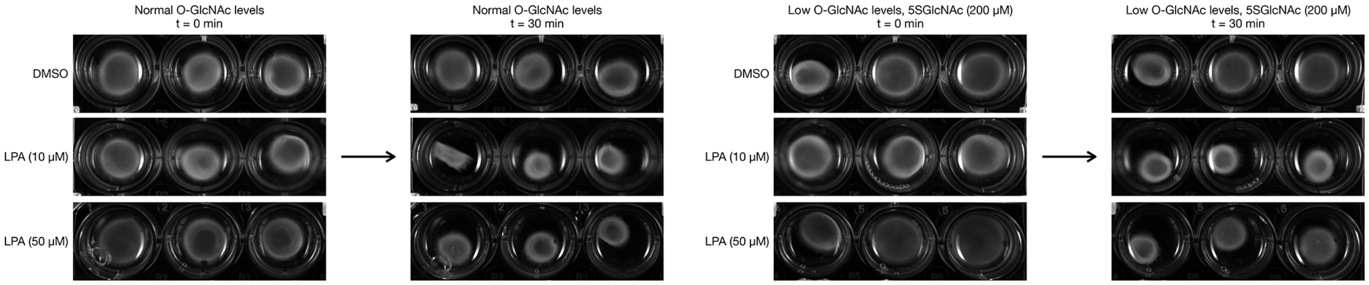

Figure S6. Raw images of the stressed collagen matrix assay. Human dermal fibroblasts cultured in stressed collagen matrices with either normal O-GlcNAc levels or low O-GlcNAc levels (5SGlcNAc, 200 μM), were treated with the indicated concentrations of LPA. The matrices were gently released and images were taken immediately (t = 0 min) and after 30 min.

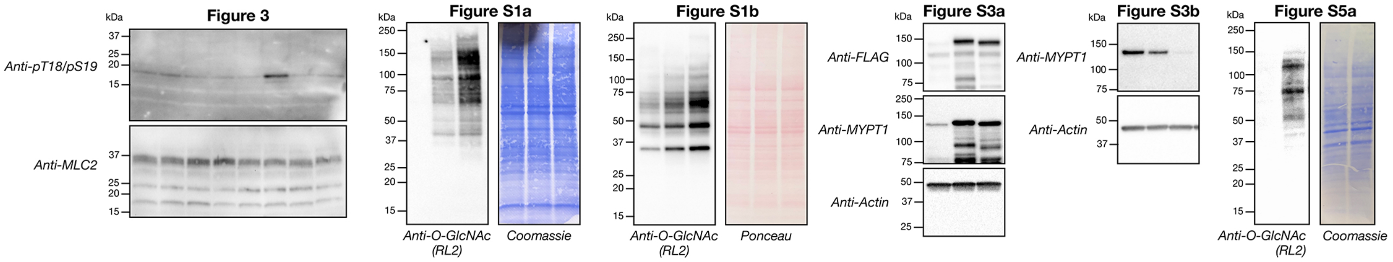

Figure S7. Un-cropped blots. Un-cropped blots from the associated main-text and supplemental figures.
